# Impact of Seasonal Variation in Pasture on Rumen Microbial Community and Volatile Fatty Acids in Grazing Yaks: Insights from High-Altitude Environments

**DOI:** 10.3390/microorganisms12081701

**Published:** 2024-08-17

**Authors:** Shichun He, Shusheng Zhao, Zibei Wang, Sifan Dai, Huaming Mao, Dongwang Wu

**Affiliations:** Yunnan Provincial Key Laboratory of Animal Nutrition and Feed, Faculty of Animal Science and Technology, Yunnan Agricultural University, Kunming 650201, China; heshichun0529@163.com (S.H.); 18487123523@163.com (S.Z.); 18487142776@163.com (Z.W.); 15987179618@163.com (S.D.)

**Keywords:** yak, volatile fatty acid, seasonal variation, microbiota, high-altitude livestock

## Abstract

The environment is one of the most important factors influencing the variation and diversity of the host gut microbiome in plateau areas. It is well-established that dietary variations substantially alter the rumen microbiota. However, there is limited research on the response of the rumen microbiota of grazing yaks to changes in seasonal diet composition under high-altitude environments. This study investigates the seasonal variations in rumen fermentation parameters, bacterial, and fungal communities in yaks, with a focus on the cold and warm seasons. Quantitative data revealed that in the cold season, yaks had an increased acetic acid proportion (*p* < 0.05) and acetic acid/propionic acid ratio (*p* < 0.05) compared to the warm season. The relative abundance of *Bacteroidetes* and *Firmicutes* were 64.67% and 25.82% in the cold season, respectively, and 66.77% and 26.87% in the warm season. The fungal community showed a higher abundance of *Ascomycetes* (58.72% to 76.91%) and *Neocallimastigomycota* in the cold season. These findings highlight the adaptation mechanisms of yaks to seasonal dietary changes and their implications for optimizing yak husbandry practices.

## 1. Introduction

Grazing yaks, native to the regions of the Tibetan plateau, have developed remarkable adaptations to survive in extreme environmental conditions characterized by low temperatures, low oxygen levels, and limited availability of vegetation [1]. Central to their adaptation is the intricate interplay between their diet, rumen microbiota, and metabolic processes, which collectively influence their health [2]. Seasonal changes in pasture composition, driven by factors such as temperature variations and precipitation patterns, profoundly affect the dietary intake of grazing yaks [3]. As herbivores, yaks primarily rely on grazing grasses and forbs for sustenance [4]. However, the nutritional content and availability of these forages fluctuate throughout the year, imposing challenges on the digestive and metabolic systems of yaks [2]. The rumen, a specialized chamber in the digestive tract of ruminant animals like yaks, harbors a diverse microbial community responsible for breaking down ingested plant materials and facilitating nutrient absorption [5]. Recent research has increasingly recognized the pivotal role of rumen microbiota in mediating the host’s response to dietary changes, influencing nutrient utilization, energy metabolism, and overall health [6]. This study aims to investigate the impact of seasonal variations in pasture composition on the rumen microbial community and volatile fatty acids (VFAs) profile in grazing yaks, particularly focusing on the northwest plateau of Yunnan Province, characterized by its high-altitude environment [7]. By employing advanced sequencing techniques such as 16S rRNA and ITS sequencing, alongside metabolomics approaches, we seek to elucidate the dynamic interactions between dietary shifts, rumen microbiota composition, and VFA production in response to changing environmental conditions [8]. Understanding how grazing yaks adapt to seasonal fluctuations in pasture quality and availability is not only essential for their welfare but also holds broader implications for sustainable livestock management in high-altitude ecosystems [9]. Insights gained from this study can inform strategies to optimize yak husbandry practices, mitigate environmental stressors, and enhance the resilience of yak populations facing the challenges of climate change and habitat degradation.

## 2. Materials and Methods

### 2.1. Experimental Animals and Sample Collection

The study was conducted at the pasture of Tiancheng Lun Zhu Agricultural Products Development Co., Ltd. in Shangri-La City (coordinates: 27°43′ N, 99°59′ E, altitude: 3600 m). The procedures were approved by the Animal Protection and Utilization Committee of Yunnan Agricultural University, China (protocol #202207030). Rumen fluid samples were collected from male yaks in May (warm season, n = 8) and November (cold season, n = 9) using a gastric tube. To avoid saliva contamination, the initial 50 mL was discarded. A total of 300 mL of rumen fluid was then collected, stored in sterile enzyme-free cryostorage tubes, and transported to the laboratory, where they were stored at −80 °C. These samples were used to determine volatile fatty acids and analyze the rumen microbial community.

### 2.2. Determination of VFA

Volatile fatty acids (VFAs) concentration was determined using gas chromatography-mass spectrometry (GC-MS). Ruminal fluid samples were placed in 1.5 mL EP tubes, to which 50 µL of 50% H_2_SO_4_ and 200 µL of an extraction agent (25 mg/L in methyl tert-butyl ether) were added. The mixture was vortexed for 30 s, oscillated for 10 min, and ultrasonicated for 10 min while incubated in ice water. Samples were centrifuged at 10,000 rpm for 15 min at 4 °C and stored at −20 °C for 30 min. The supernatant was transferred to a new 2 mL vial for GC-MS analysis using a SHIMADZU GC2030-QP2020 NX Gas Chromatography-Mass Spectrometer (San Jose, CA, USA) with an HP-FFAP capillary column. A 1 µL sample was injected at a 5:1 ratio with helium as the carrier gas. The front inlet purge flow rate was 3 mL/min, and the gas flow rate through the column was 1 mL/min. The initial temperature was 50 °C for 1 min, then increased to 150 °C at 50 °C/min for 1 min, 170 °C at 10 °C/min, 210 °C at 20 °C/min for 1 min, and finally to 240 °C at 40 °C/min for 1 min. The injection temperature was 220 °C, ion source temperature 200 °C, transfer line temperature 240 °C, and quad temperature 150 °C. The energy was −70 eV in electron impact mode. Mass spectrometry data was acquired in scan/SIM mode with the m/z range of 33–150 after a solvent delay of 3.0 min.

### 2.3. DNA Extraction, Sequencing, and Data Analysis

Genomic DNA was extracted from rumen fluid stored at −80 °C using the TIANamp Fecal DNA Kit (TianGen, Beijing, China, catalog: DP712). Normalized DNA was used for barcode PCR with specific primers. 16S rRNA/ITS genes were amplified using specific primers with barcodes. Phusion^®^ High-Fidelity PCR Master Mix (New England Biolabs, Ipswich, MA, USA) was used in PCR reactions with 2 µM forward and reverse primers and about 10 ng template DNA. The cycling conditions were: initial denaturation at 98 °C for 1 min, followed by 30 cycles of denaturation at 98 °C for 10 s, annealing at 50 °C for 30 s, elongation at 72 °C for 30 s, and a final elongation at 72 °C for 5 min. PCR products were mixed with 1X loading buffer (containing SYB green) and detected using electrophoresis on a 2% agarose gel. Products were mixed in equal density ratios and purified with a Universal DNA Purification Kit (TianGen, Beijing, China, catalog #: DP214).

Sequencing libraries were prepared using the NEB Next^®^ Ultra™ II FS DNA PCR-free Library Prep Kit (New England Biolabs, CA, USA, catalog: E7430L), incorporating indexes for sample identification. Library quantification was carried out with Qubit and real-time PCR, and size distribution was assessed using a Bioanalyzer. After pooling, libraries were sequenced on Illumina platforms. Paired-end reads were assigned to samples based on unique barcodes, and the barcode and primer sequences were trimmed. Reads were then merged using FLASH (V1.2.11, http://ccb.jhu.edu/software/FLASH/ accessed on 9 November 2023). Quality filtering was performed with fastp (Version 0.23.1) to obtain high-quality Clean Tags. Chimera sequences were detected and removed by comparing tags with reference databases (Silva for 16S; Unite for ITS) using the UCHIME algorithm.

For the effective tags obtained, denoising was performed with DADA2 or deblur module in QIIME2 [10] to obtain initial ASVs (Amplicon Sequence Variants) [11], and ASVs with abundance less than five were filtered out [12]. The database used for taxonomic assignment was the Silva database for 16S and the Unite database for ITS. To study phylogenetic relationships and differences among samples, multiple sequence alignment was performed using QIIME2 [13]. The absolute abundance of ASVs was normalized to a standard sequence number corresponding to the sample with the fewest sequences. Subsequent analyses of alpha and beta diversity were based on the normalized data. Alpha diversity was calculated from Observed_otus, Chao1, and Shannon indices in QIIME2. Beta diversity, which evaluates community composition complexity and differences between samples, was calculated based on weighted UniFrac distances in QIIME2. To study the significance of differences in community structure between groups, the adonis and anosim functions in QIIME2 were used. R software (Version 3.5.3) was used for MetaStat and T-test analysis to find significantly different species at each taxonomic level. LEfSe software (Version 1.0) was used for LEfSe analysis (LDA score threshold: 4) to identify biomarkers.

### 2.4. Statistical Analyses

Rumen VFA was compared using an independent sample *t*-test with SPSS 27.0. Results are expressed as mean ± standard deviation, with *p* < 0.05 considered significant and *p* < 0.01 considered extremely significant.

## 3. Results

### 3.1. Rumen VFA

Table 1 indicates that the content of total volatile fatty acids (TVFA) and acetic acid proportion in the warm season exceeded that in the cold season significantly (*p* < 0.01). The proportion of other fatty acids in the cold season was significantly higher than that in the warm season (*p* < 0.01). The acetic acid/propionic acid ratio was 0.73 for the cold season and 1.48 for the warm season.

### 3.2. Figures, Tables, and Schemes

**Figure 1:**
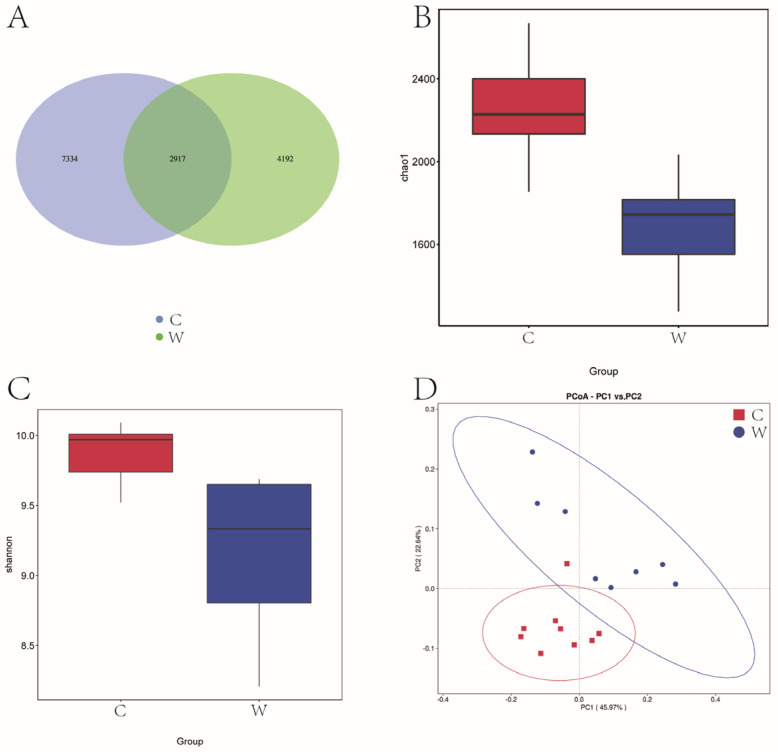
Analysis of alpha diversity and beta diversity of rumen bacteria of grazing yak in cold and warm seasons. (**A**) OTU Venn diagram. (**B**) Chao 1 index. (**C**) Shannon index. (**D**) PCoA. Cold season. (C) Warm season (W), the same as below.

Figure 1A reveals that 14,443 OTU were identified across cold and warm seasons. The C group exhibited 10,251 OTU, while the W group had 7109 OTU, and a total of 2917 OTU were present in the two experimental groups. Seasonal variations significantly influenced the Chao 1 and Shannon diversity index (Figure 1B,C). Figure 1D illustrates that yak rumen microbial communities were segregated by season in a principal component analysis (PCA), with the first two principal components (PC1 and PC2) explaining 45.97% and 22.64% of the sample variance. Cold season samples were tightly clustered, indicating lower variability, in contrast to the dispersed warm season samples, indicative of higher variability.

**Figure 2:**
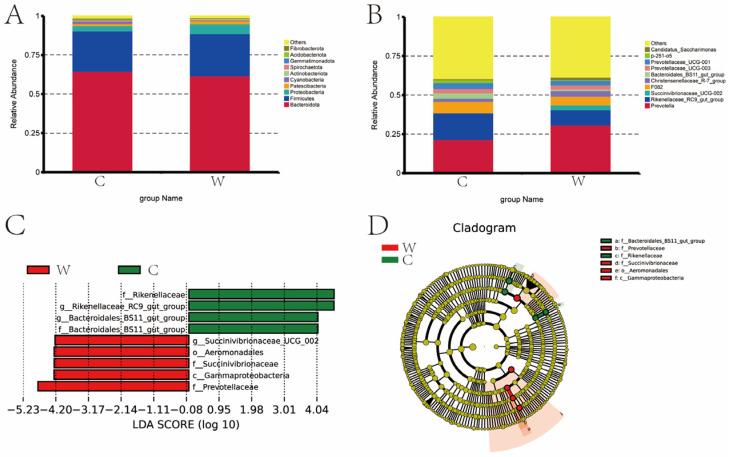
Relative abundance map of rumen bacteria of grazing yak in cold and warm seasons. (**A**) Comparison of dominant phyla in the W group and C group. (**B**) Comparison of dominant genera in the W group and C group. (**C**) LDA score. (**D**) Evolutionary branch map.

Seasonal shifts in rumen microbiota were evident in both the C and W groups (Figure 2). At the phylum level, the top 10 species in terms of relative abundance are shown in Figure 2A. *Bacteroidetes*, *Firmicutes*, *Proteobacteria*, *Cyanobacteria*, and *Patescibacteria* had higher relative abundance in the rumen, with relative abundances of 64.67% and 61.77%, 25.83% and 26.87%, 3.32% and 6.44%, 1.53% and 0.58%, and 1.41% and 0.92% in the cold and warm seasons. *Bacteroidete* and *Cyanobacteria* were higher in the cold season than in the warm season, and *Firmicutes*, *Proteobacteria*, and *Patescibacteria* were lower in the cold season than in the warm season.

Seasonal variations significantly influenced the genus-level composition of the C and W group microbiota, with *Prevotella*, *Rikenellaceae*_RC9_gut_group, *F082*, and *Christensenellaceae*_R-7_group the most abundant across seasons. The top 10 species in terms of relative abundance are shown in Figure 2B. The most abundant genus of bacteria is *Prevotella* (21.49%, 30.89%), *Rikenellaceae*_RC9_gut_group (17.21%, 9.75%), *Succinivibrionaceae*_UCG-002 (0, 3.14%), *F082* (7.30%, 5.62%), and *Christensenellaceae*_R-7_group (2.01%, 3.44%) in the cold and warm seasons. The C group is lower than the W group for *Prevotella*, *Succinivibrionaceae*_UCG-002 and *Christensenellaceae*_R-7_group, while *Rikenellaceae*_RC9_gut_group and *F082* were higher in the C group than in the W group.

Figure 2C, D present the distribution map of LDA values and the evolutionary branch map of rumen bacteria in yaks during cold and warm seasons. LEfSe analysis was used to identify seasonal bacterial signatures. In the cold season, signature rumen microbiota included Rikenellaceae, Rikenellaceae_RC9_gut_group, and Bacteroidales-BS11-gut-group. In the warm season, the key bacteria were *Succinivibrionaceae*_UCG-002, *Aeromonadale*, *Succinvibrionaceae*, *Gammaproteobacteria*, and *Prevotellaceae*.

A total of six bacteria were identified as signature microbiota for the two seasons.

**Figure 3:**
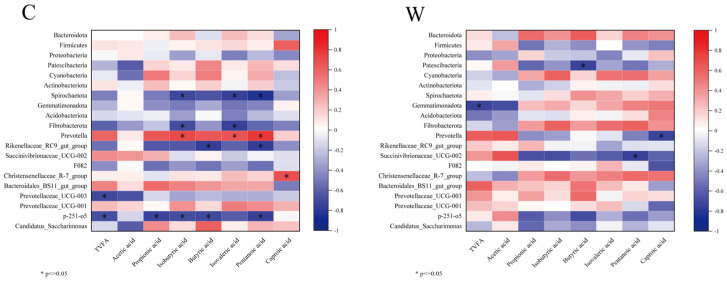
Correlation analysis of rumen fermentation parameters and bacteria, cold season (C) and warm season (W).

The 10 most abundant rumen bacterial phyla and genera were correlated with fermentation parameters, as depicted in Figure 3. In the C group, *Prevotella* showed significant positive correlations with isobutyric, isovaleric, and valeric acids (*p* < 0.05), while *Christensenellaceae*_R-7_group correlated positively with caproic acid (*p* < 0.05). Conversely, *Rikenellaceae*_RC9_gut_group and a p-215-o5 exhibited significant negative correlations with propionic acid, isobutyric acid, butyric acid, and valeric acid (*p* < 0.05). In the W group, *Prevotella* and *Succinivibrionaceae*_UCG-002 were negatively correlated with caproic and valeric acids, respectively (*p* < 0.05).

**Figure 4:**
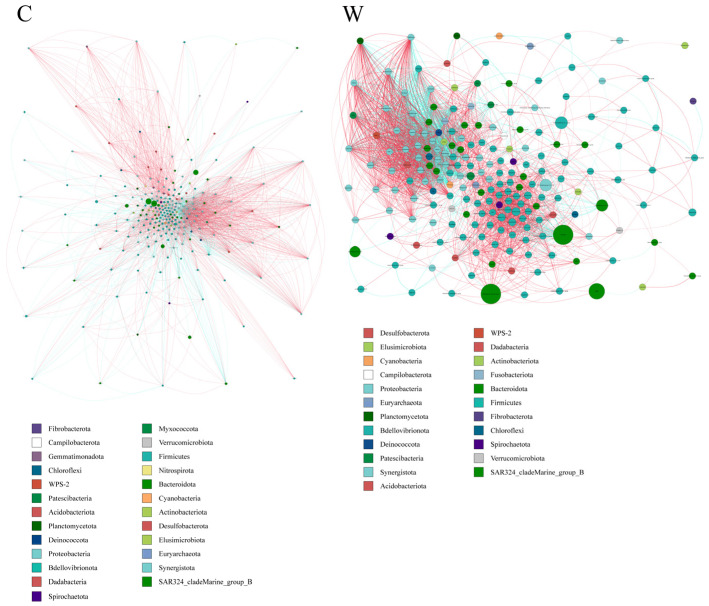
The ruminal microflora correlation network, derived from 16S rRNA gene data, reveals significant interactions with absolute correlation coefficients greater than 0.6. Node sizes represent the abundance of each taxon. Positive correlations are indicated by red lines, while negative correlations are shown in blue.

Microbial interactions in rumen bacterial communities. Microbial networks were used to analyze interactions within rumen bacterial communities in yaks. The results indicated that the cold season affected the microbial correlations (Figure 4). Specifically, negative correlations among bacterial species were found to be stronger in the warm season compared to the cold season.

### 3.3. Seasonal Variation in Rumen Fungi Composition

**Figure 5:**
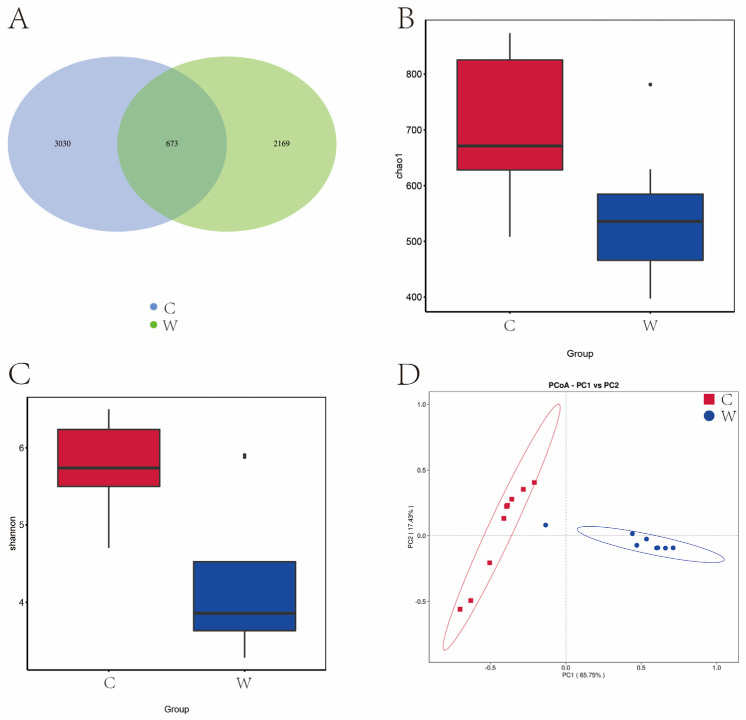
Analysis of alpha diversity and beta diversity of rumen fungi of grazing yak in cold and warm seasons. (**A**) OTU Venn diagram. (**B**) Chao 1 index. (**C**) Shannon index. (**D**) PCoA.

Figure 5A shows that a total of 5872 OTUs were identified across both cold and warm seasons. The cold season (C group) had 3703 OTUs, the warm season (W group) had 2842 OTUs, and 673 OTUs were common to both groups. The Chao 1 and Shannon indices were significantly higher in the cold season compared to the warm season (Figure 5B,C). Principal Component Analysis (PCA) revealed that PC1 and PC2 explained 66.75% and 17.43% of the variation among bacterial communities, respectively (Figure 5D).

**Figure 6:**
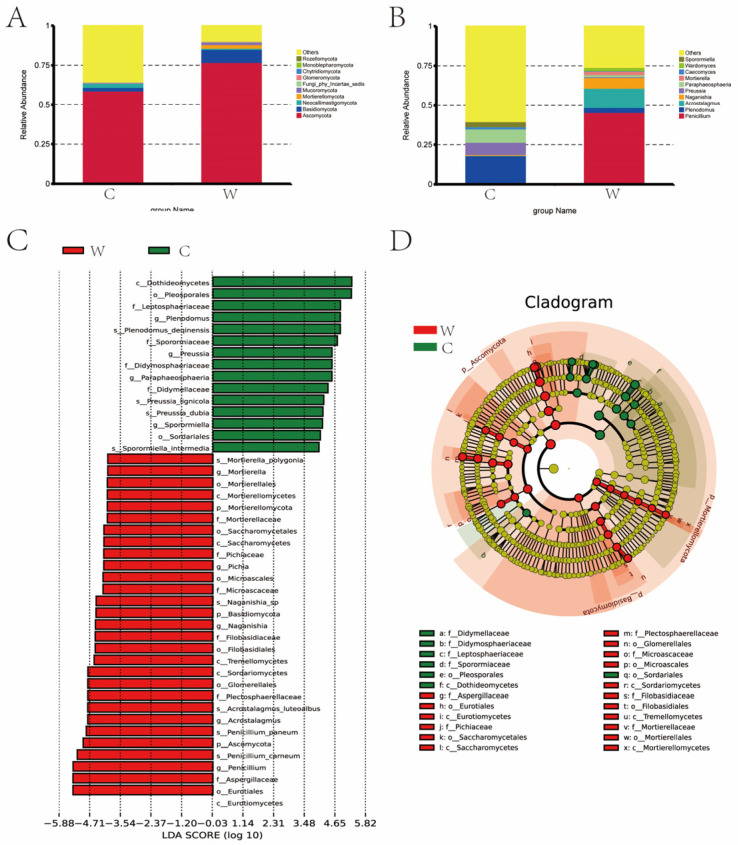
(**A**) Dominant phyla comparison: The dominant fungal phyla in the warm (W) and cold (C) seasons are compared, showing significant differences in the proportions of Ascomycota and Basidiomycota. (**B**) Dominant genera comparison: This comparison highlights the top fungal genera in both the W and C groups, noting significant seasonal variations in their relative abundances. (**C**) LDA Score: The LDA score distribution identifies the key fungi that distinguish the two seasons, highlighting 24 signature fungi across both groups. (**D**) Evolutionary branch map: This map illustrates the phylogenetic relationships and evolutionary differences among the dominant fungal species in the rumen fluid during cold and warm seasons.

The abundance of fungi varied significantly between seasons, with greater diversity observed in the cold season (C group), as indicated by the Chao 1 and Shannon indices. Figure 6A shows that Ascomycota and Basidiomycota were the dominant fungal phyla, with proportions of 58.72% and 76.91% in the cold season, and 2.35% and 8.10% in the warm season, respectively. In the cold season, *Neocallimastigomycota* (2.56%) was notable, while in the warm season, *Mortierellomycota* (2.31%) and *Mucoromycota* (1.61%) were more prominent. Figure 6B lists the top ten fungal genera with their relative abundances across both seasons. In the cold season *Penicillium* (0.94%, 45.52%), *Plenodomus* (17.15%, 3.25%), *Acrostalagmus* (0.17%, 12.11%), *Naganishia* (0.58%, 6.73%), *Preussia* (7.74%, 0.60%), *Paraphaeosphaeria* (8.47%, 1.29%), *Mortierella* (0.03%, 2.22%), *Caecomyces* (1.39%, 0.33%), *Wardomyces* (0.06%, 1.89%), *Sporormiella* (3.21%, 0.08%) were more abundant. In the warm season *Penicillium*, *Acrostalagmus*, *Naganishia*, *Mortierella*, and *Wardomyces* were more abundant compared to the cold season. Figure 6C,D depict the LDA value distribution and evolutionary branch map of rumen fungi. A total of 24 fungi were identified as signature microbiota for the two seasons. In the cold season Didymellaceae, *Didymosphaeriaceae*, *Leptosphaeriaceae*, *Sporormiaceae*, *Pleosporales*, *Dothideomycetes*, and *Sordariale* were identified. In the warm season *Aspergillaceae*, *Eurotiales*, *Eurotiomycetes*, *Pichiaceae*, and 13 other species were identified.

**Figure 7:**
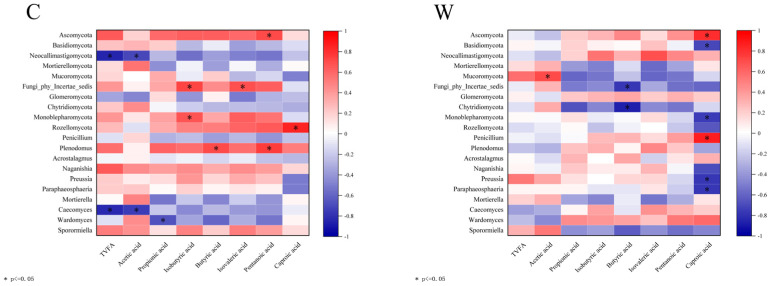
Correlation analysis of rumen fermentation parameters and fungi.

Correlation analysis of rumen fungi and rumen fermentation parameters is shown in Figure 7. In the C group, *Plenodomus* correlated positively with butyric and valeric acids (*p* < 0.05), while *Caecomyces* showed a negative correlation with TVFA and acetic acid (*p* < 0.05). Preussia and paraphaosphaeria were negatively correlated with caproic acid in the W group (*p* < 0.05).

**Figure 8:**
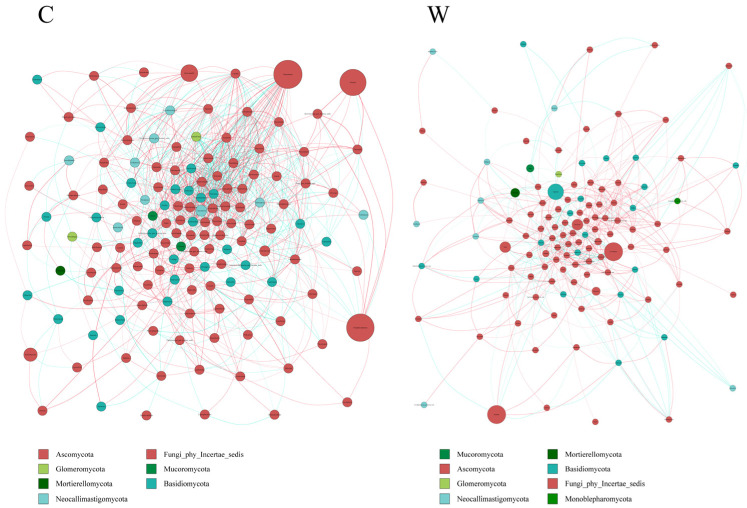
Interaction network of ruminal fungi. The ruminal microflora correlation network, based on ITS gene data, revealed statistically significant interactions with absolute correlation coefficients greater than 0.6. Node sizes correspond to the abundance of each taxon. Positive correlations are represented by red lines, while negative correlations are shown in blue.

Analysis of rumen fungi communities in yak microbial networks (Figure 8) indicated that cold seasons altered the correlation within the microbiota. These correlations were more pronounced in the cold than in the warm seasons, with a predominance of positive over negative associations. The cold season was characterized by a more diverse fungi composition and the presence of key microorganisms.

## 4. Discussion

### 4.1. Effect of Rumen Fermentation Parameters of Yaks in Cold and Warm Seasons

Volatile fatty acids (VFA), crucial for ruminant nutrition and health, are generated by rumen microbes fermenting cellulose and other carbohydrates [14]. Acetic acid, propionic acid, and butyric acid are the main components of volatile fatty acids in rumen fluid [15]. Acetic acid is one of the key substances in the body for fat synthesis, propionic acid is an important raw material for gluconeogenesis, and butyric acid can be absorbed and converted to beta-hydroxybutyric acid to provide energy for muscle tissue and to synthesize fatty acids by other biochemical pathways. The proportion of carbohydrates in the diet can affect the levels of volatile fatty acids [16]. As the seasons change from warm to cold, and from wet to dry, the fermentable carbohydrate content of the forage decreases while the fiber content increases. In this case, yaks suffer from a variety of nutritional deficiencies. In this study, the proportion of acetic acid and TVFA in yaks is higher during the warm season, which may be due to the high crude fiber content of winter forage, which is difficult to digest, and the inadequate supply of nitrogen sources. The structural carbohydrates in the diet are fermented by fiber-degrading bacteria to produce volatile fatty acids with a lower content. In conclusion, due to seasonal differences in forage yield and nutritional quality, volatile fatty acids produced by rumen fermentation were lower in the cold season than in the warm season.

### 4.2. Effects of Rumen Bacteria of Yaks in Cold and Warm Seasons

Our research confirms that *Bacteroidetes* and *Firmicutes* are the predominant phyla in the rumen microbiota of treated yaks, aligning with studies on beef cattle, dairy cattle, and sheep [17,18,19]. Together, they constitute approximately 80% of the microbiota, overshadowing less abundant phyla such as *Fibrobacter*, *Spirochaeta*, and *Proteobacteria*, which is consistent with prior research findings [20,21]. Notably, *Bacteroidetes* were more prevalent than *Firmicutes* in our study, echoing findings from another yak microbiota investigation [22].

In the rumen, *Bacteroidetes* and *Firmicutes* accounted for 64.67% and 25.82% during the cold season, respectively, and 66.77% and 26.87% in the warm season. It was found that *Bacteroidetes* dominated the rumen of ruminants, which play an essential role in the breakdown and utilization of carbohydrates, polysaccharides, and proteins in the diet. The relative abundance of *Firmicutes* increases with increasing forage quality. Therefore, in this study, *Firmicutes* have a high content in the W group. *Firmicutes* possess numerous genes encoding enzymes involved in energy metabolism. They produce various digestive enzymes that break down different substances, aiding the host in nutrient digestion and absorption. *Bacteroidetes* are mainly involved in carbohydrate fermentation as well as polysaccharide, protein, and bile acid substitution, contributing to enhanced nutrient utilization and immune function in the host. Grazing yaks face reduced nutrient intake during the cold season compared to the warm season. The enrichment of *Bacteroidetes* during the yaks’ cold season helps extract carbohydrates and other nutrients from the harsh environment to meet winter energy requirements and plays an important role in improving body immunity.

*Prevotella* is the dominant genus in the rumen, consistent with findings in beef cattle, dairy cattle, and goats from previous studies [23,24,25]. The genus *Prevotella* secretes a variety of enzymes such as proteases, amylases, and hemicellulases to break down protein, starch, pectin, and hemicellulose in the diet [26]. *Prevotella*, *Butyrivibrio*, *Fibrobacter*, *Rikenellaceae*_RC9_gut_group and *Bacteroidales*_ BS11_gut_group_norank and unclassified bacteria were the dominant genera in the rumen bacterial community [27]. In addition to the *Fibrobacteria*, which are generally considered to have fiber-degrading functions, the *Firmicutes* and *Spirochaetes* have also been shown to be associated with the degradation of high-fiber forages [28]. In this study, the rumen flora of each yak responded to seasonal changes with differences in the relative abundance of hydrolysate-degrading bacterial genera such as *Prevotella*, *Rikenellaceae*_RC9_gut_group, *Succinivibrionaceae*_UCG-002 at the genus level.

### 4.3. Effects of Rumen Fungi of Yaks in Cold and Warm Seasons

Fungi are an important component of rumen microorganisms, and fungi degrade polysaccharides such as cellulose and starch in feed to produce large amounts of H_2_, CO_2_, formate, and acetate as metabolites [29]. In addition, the initial colonization of fibers by rumen anaerobic fungi can promote rapid fibrinolytic activity by bacteria and other microorganisms, and fiber degradation can influence the composition of other microbial communities [30]. Compared to other rumen microorganisms, fungi are an important source of cellulases, ligninases, and other hydrolase enzymes which play a key role in the degradation of lignocellulose in the rumen [31,32]. Therefore, rumen fungi are considered primary intruders that initiate the degradation of fiber feed particles [33]. This study found that the higher abundance of fungi in the cold season may be related to the higher lignin content of forage in the cold season. *Ascomycetes* and *Neocallimastigomycota* were the predominant phyla in the rumen. *Ascomycetes* are a diverse group of more than 3000 species, including yeasts. *Ascomycetes* contain chitin in the cell wall and produce non-flagellated spores. Except for some yeasts, which do not form hyphae, all ascomycetes are mycelial. In the present study, 58.7% to 76.9% of the ascomycetes were finally detected in the rumen fluid samples. *Neocallimastigomycota* in the rumen has fiber-degrading enzyme activity, which can effectively degrade fiber and other substances in the diet and improve the utilization rate of roughage [34]. The relative abundance of *Preussia* in the rumen of yaks was significantly higher in the cold season than in the warm season, which may be an endemic genus formed in the grazing environment of yaks. The alpha diversity index shows that the diversity of rumen fungi is lower in summer than in winter, with significant differences in abundance. This may be due to changes in diet and environment. In the warm season, pasture grasses are juicy, palatable, and more nutritious, and the number of bacteria in the rumen increases, and a large number of bacteria have been shown to produce fungal inhibitors that inhibit fungal growth. It is worth noting that penicillium changed from 0.94% in the cold to 45.52% in the warm season. Implications of the dramatic change in penicillium include: (1) Adaptation to temperature: The significant increase in *Penicillium* during the warm season may indicate that this genus thrives in higher temperatures. *Penicillium* species are known for their ability to grow in diverse and sometimes extreme environments, which might give them a competitive advantage as temperatures rise [35]. (2) Nutrient availability: Seasonal changes in diet due to the availability of different types of forage could alter the nutrient composition in the rumen. The warm season typically provides a more abundant and varied plant diet, which might favor the growth of *Penicillium*. This genus is known for its capacity to decompose a wide range of organic materials, suggesting that the nutritional profile of the rumen in warmer months supports its proliferation [36]. (3) Metabolic impact: The dramatic increase in *Penicillium* may have metabolic implications for the host. *Penicillium* species can produce a variety of secondary metabolites, including enzymes that degrade complex plant polymers. This enzymatic activity could enhance the breakdown of plant material in the yak’s diet, potentially improving nutrient absorption and energy efficiency [37]. (4) Potential pathogenicity: While many *Penicillium* species are benign or beneficial, some can be pathogenic or produce mycotoxins. The rise in *Penicillium* levels raises questions about the potential risks associated with higher concentrations of these fungi. Investigating whether the specific *Penicillium* species present are harmful or beneficial to the yaks’ health is crucial [38]. (5) Environmental indicators: The change in *Penicillium* composition serves as an indicator of broader environmental shifts. Understanding these microbial dynamics can provide insights into how climate change and seasonal variations affect livestock health and productivity. It highlights the importance of monitoring microbial communities as part of ecological and agricultural management strategies [39]. At different times of the year, grazing yaks consume forages with different nutrient contents, which affects not only the number and relative proportion of rumen microbiota but also the microbiota and its diversity.

This study underscores the significant impact of seasonal variations on the rumen microbiota of yaks, highlighting the need for targeted nutritional and management strategies to optimize yak health and productivity. The findings suggest that dietary adjustments according to seasonal changes can enhance nutrient utilization and immune function in yaks. Future research should explore the functional implications of these microbial changes and develop interventions to enhance the sustainability of yak farming practices. Additionally, investigating the potential benefits of supplementing yaks’ diets with specific nutrients or probiotics during different seasons could further improve their health and productivity.

## 5. Conclusions

The rumen microbial community structure of grazing yaks may undergo a significant shift during the cold season, possibly reflecting the microbial community’s rapid adaptation to changes in nutrient and substrate availability due to seasonal variations. This study furthers our understanding of how microbial adaptation to seasonal variations in nutrient availability and climate may function in high plateau ruminants.

## Figures and Tables

**Table 1:** Effects of cold and warm seasons on the rumen VFA of yaks.

Item	C	W	*p*-value
Total volatile fatty acids (mM)	18.37 ± 1.97	22.90 ± 3.38	<0.01
Acetic acid (%)	25.30 ± 11.31	44.23 ± 9.51	<0.01
Propionic acid (%)	38.11 ± 6.59	31.44 ± 5.09	0.02
Isobutyric acid (%)	2.80 ± 0.52	1.99 ± 0.40	0.001
Butyric acid (%)	28.76 ± 3.43	20.02 ± 4.30	<0.01
Isovaleric acid (%)	1.99 ± 0.36	1.05 ± 0.32	<0.01
Pentanoic acid (%)	2.24 ± 0.49	1.16 ± 0.27	<0.01
Caproic acid (%)	0.80 ± 0.21	0.12 ± 0.05	<0.01
Acetic acid/Propionic acid	0.73 ± 0.46	1.48 ± 0.55	0.006

Note: Cold season (C) and warm season (W), the same as below.

## Data Availability

The datasets presented in this study can be found in online repositories. The names of the repository and accession number(s) can be found in the NCBI SRA database with accession numbers PRJNA1036001, and PRJNA1034800.
